# Editorial: Mesenchymal Stem Cells and Interactions With Scaffolds - Biomaterials in Regenerative Medicine: From Research to Translational Applications

**DOI:** 10.3389/fcell.2019.00193

**Published:** 2019-09-12

**Authors:** Francesco De Francesco

**Affiliations:** Hand Surgery Unit, Department of Plastic Reconstructive Surgery, Azienda Ospedaliero Universitaria Ospedali Riuniti, Ancona, Italy

**Keywords:** regenerative medicine, adipose stem cells, scaffold, translational applications, mesenchymal stem cells

It can be estimated that 1,500,000 patients in Europe undergo soft tissue reconstruction each year, and of these patients, about 20% undergo an experience of a loss of function despite reconstruction and, about 30,000 patients in Europe are suffering from donor site morbidity relating to flap reconstructions. In a defect, the different missing tissues involved have specific functions and their replacement is often quite difficult. For example, the closure of a defect is commonly associated with the transfer of tissue (e.g., a flap), which may not fully restore the unique function of the lost part. Also, each tissue transfer is associated with donor site morbidity, the most important being scars, infection and loss of function.

The ability to regenerate tissues is attributable to a pool of undifferentiated cells capable of replacing damaged cells in order to guarantee the integrity of the organism. The main source of stem cells from adult is the bone marrow (BMSC, Bone Marrow Stromal Cells) can easily be obtained from its stroma. A particularly interesting source of mesenchymal stem cells is represented by stem cells that can be isolated from adipose tissue (ASCs, Adipose Stem cells; Zuk et al., 2002; De Francesco et al., 2009). The ASCs have the potential to differentiate into bone (Tajma et al., 2018), cartilage (Szychlinska et al., 2017), skeletal muscle (Desiderio et al., 2013), fat (Ferraro et al., 2012), and other tissue (Planat-Benard et al., 2004; Tobita et al., 2008) when cultivated under specific conditions. The mesenchymal cells of adipose tissue are similar to bone marrow in the treatment of various tissue pathologies and therefore represent an important source for autologous cellular therapies. Fellows et al. explore the challenges associated with cartilage repair and regeneration using MSC-based cell therapies, focusing on cells capable of producing stratified hyaline-like articular cartilage regeneration. Moreover, Senesi et al. stated that ASCs play an important role in the treatment of osteoarthritis and the authors compare the mechanical and exymathic procedure to isolate the stromal vascular fraction from adipose tissue. In this paper, the authors showed that the mechanical procedures yielded no significant difference in cell viability and cluster differentiation expression to enzymatic procedure. Currently, the use of mechanical procedures for the isolation of the SVF from adipose tissue has become of vital importance. Especially for the requirements of minimal tissue manipulation and the impossibility of using collagenase. About that, Dessels et al. stated that ASCs must be used with good manufacturing procedures (GMPs) in humans and therefore fetal bovine serum, normally used *in vitro* research, can not be used. Then, for clinical trial research, ASCs must be expanded *in vitro* using xeno-free products and human blood-derived alternatives. In addiction, Purpura et al. collected adipose tissue using a water-jet assisted liposuction in order to preserve an high cell viability for the immediate use in the field of plastic and reconstructive surgery.

In addition to ASCs, the large family of mesenchymal stem cells also includes cells from oral and dental tissue. In this context, human periapical cysts mesenchymal stem cells (hPCy-MSCs) have been studied by Tatullo et al. This cells exhibit extensive proliferative potential, cell surface marker profile, and the ability to differentiate into various cell types.

Tissue engineering (TE), a collection of technologies combining biomaterials and stem cells, provides the tools for regenerative medicine (RM) and is expanding tremendously from biomaterial science toward a genuine multidisciplinary area, integrating biology, medicine, and various engineering sciences (Langer and Vacanti, 2016). Rodriguez y Baena et al. evaluated, in this paper, the ability of autologous periosteum-derived micrografts in combination with a new biomaterials such as poly(lactic-co-glycolic acid) (PLGA) supplemented with hydroxyl apatite (HA) for bone augmentation in the sinus lift procedure. In this manner, the authors showed an increased percentage of vital mineralized tissue in the treated group respect to the control, confirmed by histological analysis and Rx evaluation. The use of biomaterial scaffolds selected in an appropriate way, together with growth factors can significantly improve survival and differentiation of transplanted stem cells. The biomaterial/scaffold itself, actually, may be an ideal delivery vehicle of chemical factors.

We know from these studies and from many other published studies, that the MSCs have been used in a significant number of clinical trials with a good regenerative effect. Despite this, the functional role of these cells in tissue regeneration is not yet fully understood. In a review, Lunyak et al. demonstrated that MSCs have an autocrine and paracrine properties on the regulation of immune system and induce changes in the tissue microenvironment with numerous therapeutic effects on promoting repair and regeneration.

The current Research Topic will focus on the regeneration, repair, and rebuilding of tissues combining implantable biocompatible materials with stem cell technologies. Although numerous breakthroughs in stem cell research have been made thus far, their success and applicability in clinical trials remains to be ascertained. A number of issues will be important for the advancement of regenerative medicine as a field. First, stem cells, whether isolated from adult tissue or induced, will often require tight control over their behavior to increase their safety profile and efficacy after transplantation. Second, the creation of microenvironments, often modeled on various stem cell niches that provide specific cues, including morphogens and physical properties, or have the capacity to genetically manipulate target cells, will likely be key to promoting optimal regenerative responses from therapeutic cells. In the last decade several skills were acquired about the isolation, the morphological characteristics and the differentiation potential of adult stem cells *in vitro*.

## Author Contributions

FD contributed conception and design of the study, wrote the first draft of the manuscript, wrote sections of the manuscript, read, and approved the submitted version.

### Conflict of Interest Statement

The author declares that the research was conducted in the absence of any commercial or financial relationships that could be construed as a potential conflict of interest.
